# Current capabilities and future perspectives of FCS: super-resolution microscopy, machine learning, and in vivo applications

**DOI:** 10.1038/s42003-023-05069-6

**Published:** 2023-07-07

**Authors:** Jagadish Sankaran, Thorsten Wohland

**Affiliations:** 1 Genome Institute of Singapore, Agency for Science, Technology and Research, Singapore, 138632 Singapore; 2 Department of Biological Sciences, National University of Singapore, Singapore, 117558 Singapore

**Keywords:** Imaging, Cellular imaging

## Abstract

Fluorescence correlation spectroscopy (FCS) is a single molecule sensitive tool for the quantitative measurement of biomolecular dynamics and interactions. Improvements in biology, computation, and detection technology enable real-time FCS experiments with multiplexed detection even in vivo. These new imaging modalities of FCS generate data at the rate of hundreds of MB/s requiring efficient data processing tools to extract information. Here, we briefly review FCS’s capabilities and limitations before discussing recent directions that address these limitations with a focus on imaging modalities of FCS, their combinations with super-resolution microscopy, new evaluation strategies, especially machine learning, and applications in vivo.

## Abstract (web summary)

The Mini Review discusses recent developments in fluorescence correlation spectroscopy (FCS) and combinations of FCS with super-resolution imaging and machine learning.

## Introduction

**Fig. 1:**
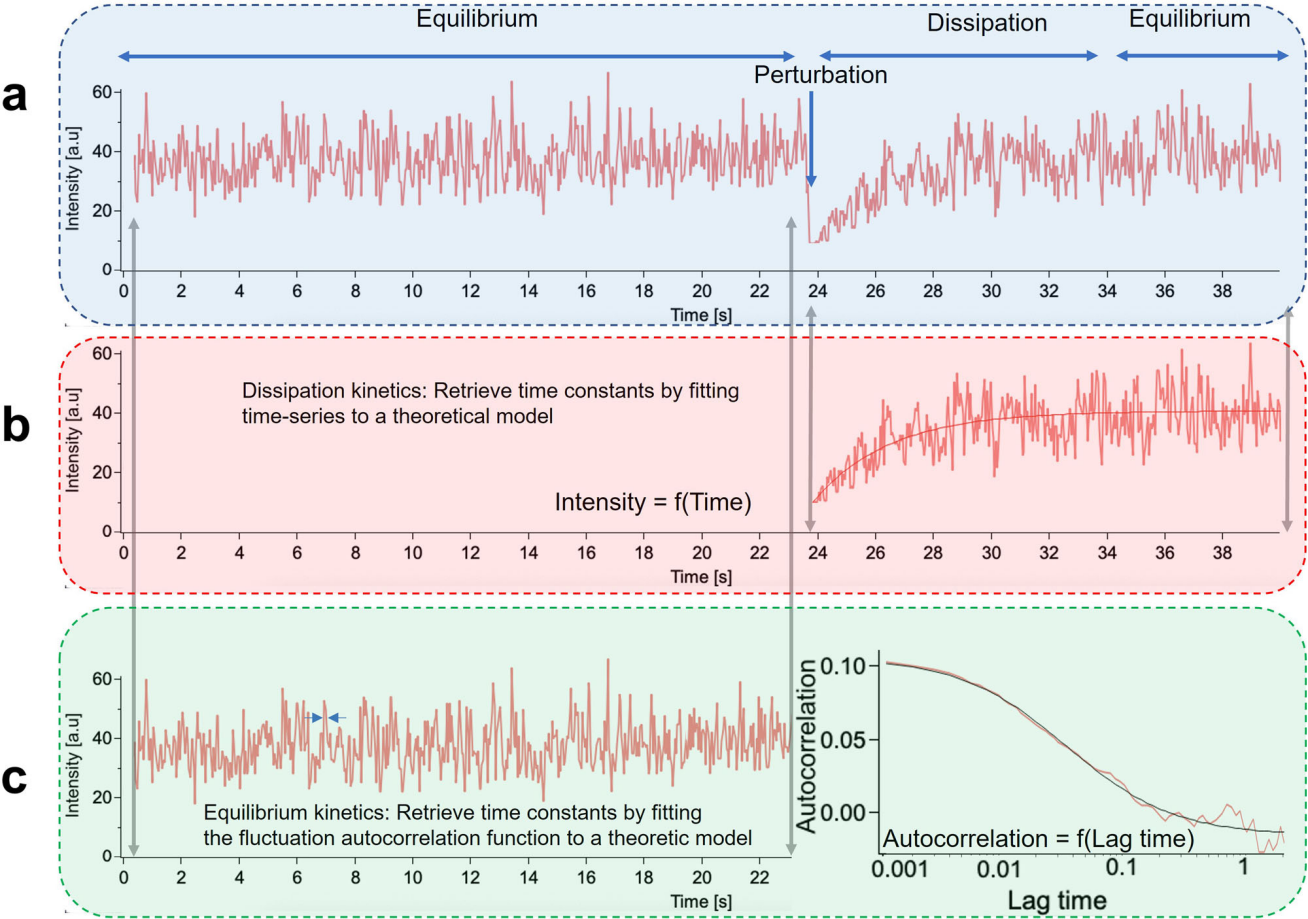
Perturbation versus spontaneous fluctuation analysis:. **a** The kinetics of a process in equilibrium is quantified by two different methods-invasively, by intentionally forcing the system away from equilibrium using an external stressor (perturbation analysis) or non-invasively by analyzing the spontaneous deviation from equilibrium (fluctuation analysis). **b** In relaxation analysis, one characterizes the time needed for the system to dissipate the effect of the perturbation, i.e., to return to the original equilibrium state, by fitting a theoretical model (smooth solid line) to compute the characteristic time constant. **c** In equilibrium kinetics, the characteristic time of fluctuations around the equilibrium (left) is typically examined using temporal autocorrelation analysis (right). To this aim, the autocorrelation function is computed for different lag times and plotted as a function of lag time to yield an autocorrelation curve (red), which is then fitted using a theoretical model (black) to determine the characteristic decay time of the autocorrelation curve.

The year 2022 marks the golden anniversary of the first paper^1^ describing the fluctuation-based spectroscopic technique called fluorescence correlation spectroscopy (FCS). FCS is based on the fluctuation dissipation theorem and provides information about equilibrium and reaction kinetics that could previously only be obtained by the various relaxation techniques pioneered by Manfred Eigen^2,3^. In relaxation techniques, the return to equilibrium of a reaction system whose temperature, pressure or electric field was perturbed provides information about the system’s reaction kinetics (Fig. 1).

Fluctuations are spontaneous deviations from equilibrium that contain information about the system’s relaxation to equilibrium and thus their analysis carries the same information as the perturbation experiments. Therefore, without any external perturbation, FCS exploits the information in fluctuations around the equilibrium state to understand the kinetics of the system. Apart from being non-invasive, the use of fluorescence as the fluctuating quantity to monitor the equilibrium provided better specificity and sensitivity over other fluctuation monitoring techniques including scattering. Detailed descriptions of the theory^4^, experimental realization^5^, and statistical accuracy^6^ of FCS have been published earlier.

Fluctuations in fluorescence intensity are defined mathematically as deviations from the mean fluorescence intensity. For systems in equilibrium, the mean fluctuation in fluorescence intensity is zero and hence is a challenging physical parameter to analyze and interpret. As a result, a convenient way to analyze fluctuations is to use autocorrelation functions which measure the self-similarity, of the fluctuating signal. Typically, while performing an FCS experiment, the autocorrelation function of the detected fluorescence is first computed for different lag times. Then, the computed function is approximated by a suitable theoretical model^7^ to determine the underlying physical parameters of the fluctuating signal.

The initial FCS experiments were plagued by high background, limited computational capabilities and long measurement times. The reduction in background by the use of confocal based detection in FCS led to a breakthrough in the field^8^, which manifested as a ten-fold increase in number of papers per year compared to the preceding decade. The commercialization of FCS by Evotec and Zeiss made it widely available and sparked an increase in FCS applications especially in biology^9^.

The widespread use of confocal microscopy in biology and the ability to perform FCS in live cells rendered FCS a suitable technique for investigating various physicochemical phenomena observed in cell^10^ and developmental biology^11–13^. FCS has been used to investigate diffusion, convection, chemical kinetics^14^, affinities^15^ for binding to both immobile and mobile structures, concentrations, stoichiometry, or the microscale organization of cell-membranes^16^. An overview of the versatility of FCS can be gained from various reviews on the topic^17–20^. Apart from standard FCS, scanning FCS (line-scan^21^ or circular^22^) refers to the group of techniques where the measurement volume is moved across the sample and is very useful for investigating slow diffusion on membranes. The interested reader is referred here for a detailed description of scanning FCS^23^.

Measurements described so far enabled the quantification of dynamics at only one confocal diffraction limited spot at a time. Any attempt at covering larger areas required scanning the sample one spot at a time which proved to be time consuming, led to asynchronous measurements and was prone to photobleaching. Raster Image Correlation Spectroscopy (RICS) improved on this situation by using fast scanning and by utilizing the intrinsic time structure of the scanning process to combine spatial and temporal correlations^24,25^. As RICS can be implemented at any available confocal microscope it is widely accessible^26,27^. Typically employed as a single point method, current implementations of RICS enable multiplexed detection leading to creation of diffusion maps^27–29^. Further advances were made using multiple confocal spots with multiple detectors^30^ or detectors with multiple elements^31–33^ which have culminated in so-called massively parallel FCS^34–36^.

Alternatively, spatial fluctuations were utilized in Image Correlation Spectroscopy (ICS)^37,38^ to investigate clustering and aggregation of molecules or molecular complexes, even those with sizes well below the diffraction limit, initially not including dynamics. Later, ICS measurements were also collected in time to evaluate the temporal development of spatial correlations^39,40^.

**Fig. 2:**
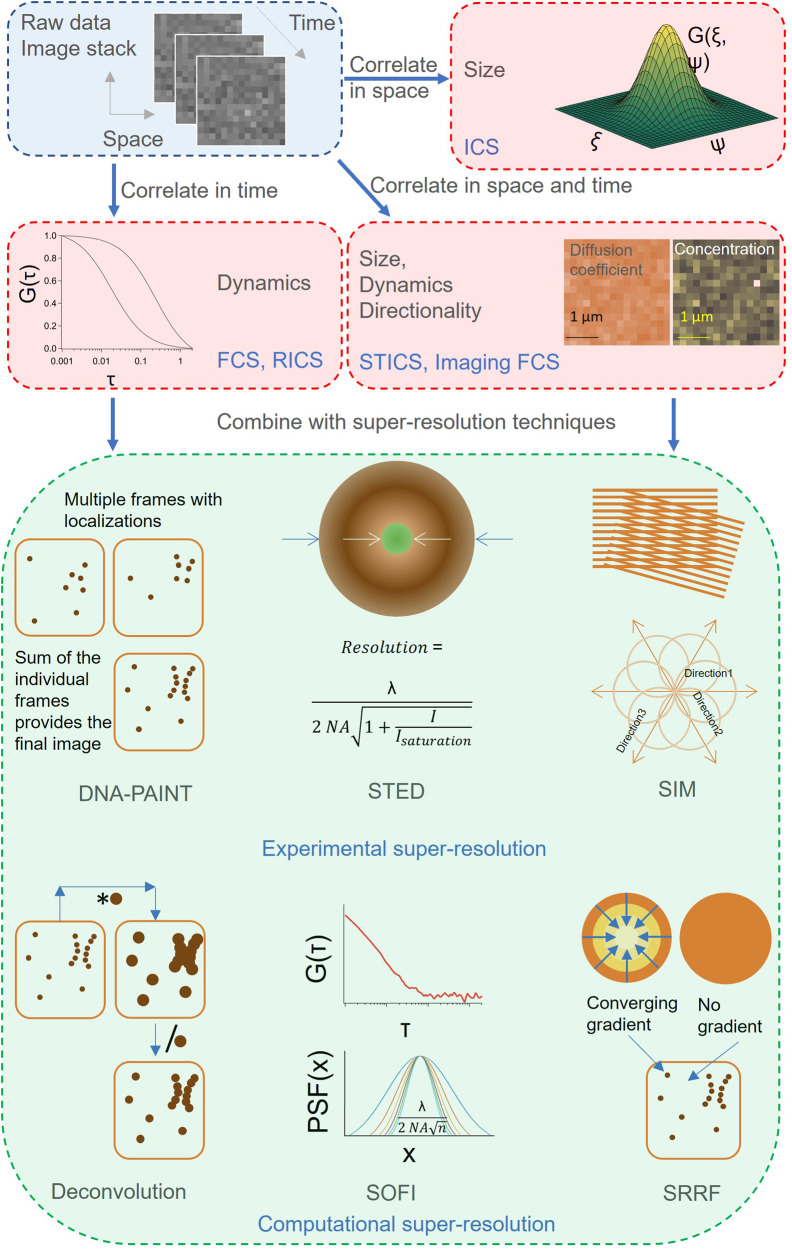
Schematic of current correlation-based techniques. Correlation-based techniques are classified based on whether they evaluate spatial, temporal, or spatiotemporal correlations. ICS utilized spatial correlations to investigate clustering and aggregation of molecules or molecular complexes. FCS utilizes temporal correlations to investigate diffusion, convection, chemical kinetics, affinities. RICS utilizes the intrinsic time structure of the scanning process to obtain the correlations. Spatiotemporal analysis of image stacks is performed in STICS and Imaging FCS. Current methodologies making use of either point or array detection have accordingly been combined with various super-resolution techniques. DNA-PAINT involves labeling single molecules with fluorescently tagged DNA transiently binding to target sequences on the sample. Sequential detection and precise localization of single molecules result in super-resolved images. STED involves depleting the fluorescence at the fringes of the excitation beam by stimulated emission leading to detection in sub-diffraction limited volumes in the sample. SIM involves illuminating the sample with structured illumination patterns leading to detection of spatial frequencies in an image that would otherwise be below the diffraction limit. Deconvolution microscopy is a technique that reverses the blurring effects of the point spread function of the microscope on the images. SOFI calculates correlation functions, in which the PSF appears in higher powers, resulting in improved resolution. SRRF determines the convergence point of the radial gradients of light distributions to estimate the localization of the source of the emission.

The true simultaneous analysis of spatial and temporal^41^ correlations over whole images was made possible by the introduction of fast and sensitive array detectors (EMCCD^42–45^, sCMOS^31,46,47^, and SPAD arrays^48,49^). The spatiotemporal analysis of image stacks is referred to as spatiotemporal ICS (STICS) or Imaging FCS (Fig. 2).

Apart from multiplexing, i.e., the recording of multiple simultaneous measurements, Imaging FCS allows calculating all spatiotemporal correlations between any pixels or group of pixels^50^. Therefore, a single measurement contains the data for the analysis of the dynamics over many length scales by pixel binning or by choosing any point of interest and analyze their relations. It also avoids sampling bias by recording and quantifying the heterogeneity in an entire cell^51^. Other useful methods to quantify heterogeneity are pair-correlation functions^52,53^ and differences in the spatial correlation functions of adjacent pixels (ΔCCF)^50^.

This capability of analyzing fast molecular dynamics over whole images with single molecule sensitivity at physiologically relevant concentrations made FCS an attractive tool for combination with super-resolution techniques, which provide superior structural details but at much lower time resolution. This combination of recording simultaneously fast dynamics and super-resolution images was exploited either by combinations of correlation spectroscopies with experimental or computational super-resolution techniques.

### Combination of FCS and super-resolution

FCS has been combined with experimental and computational super-resolution techniques, leading to improved simultaneous spatiotemporal resolution. On the experimental super-resolution front, correlation spectroscopies have been integrated with stimulated emission depletion microscopy (STED)^54^, DNA points accumulation for imaging in nanoscale topography (DNA-PAINT)^55^, structured illumination microscopy (SIM)^56^, and airyscan units^57^.

In STED, fluorophores are selectively depleted at certain regions to improve the resolution providing access to measure diffusion and concentrations on length scales as small as 30 nm^54^. This was employed to distinguish free and anomalous diffusion due to transient binding. A combination of FCS with DNA-PAINT called localization-based FCS^55^ (lbFCS) enables the quantification of absolute number/concentration of molecules. SIM, which applies low laser powers with standard fluorophores using patterned light sources in live cells, was combined with STICS^56,58,59^ to measure transport velocities. As STICS calculates spatial correlations, the better resolution of SIM resulted in better-resolved velocity parametric maps. The combination of STICS with SIM has also been extended to cross-correlations^60^. By utilizing two different markers localized on cell-membrane and cytoskeleton, correlations of the velocity parametric maps demonstrated coupling between the flows of the cell-membrane and cytoskeleton.

On the computational side, correlation spectroscopies have been combined with super-resolution optical fluctuation imaging (SOFI)^47,61–63^, super-resolved radial fluctuation (SRRF)^47^, and deconvolution^47^, with other potential techniques being mean-shift super-resolution (MSSR)^64^, sparsity-based super-resolution correlation imaging (SPARCOM)^65^, or multiple signal classification algorithm (MUSICAL)^66^.

In super-resolved optical fluctuation imaging (SOFI)^61^, second, fourth, or even higher order autocorrelation functions are calculated on time-traces of fluorescent molecules which show blinking behavior. As a result of higher order autocorrelation analysis, the point spread function (PSF) is reduced leading to an improvement in resolution. Typically, an *n*^th^ order correlation leads to a $\sqrt{n}$ improvement in resolution^61^. Super-resolution radial fluctuations (SRRF)^47^ microscopy is a super-resolution technique that performs a SOFI analysis on radiality stacks.

SOFI^47,62^ and SRRF^47^ are computational super-resolution techniques and thus no hardware add-ons are necessary to be installed with the microscope. They can be thus applied to the exact same data as Imaging FCS. However, these techniques need different acquisition strategies. Hence the initial raw imaging data is collected at the best experimentally accessible spatiotemporal resolution. The data is rescaled in space or time depending on the needs of the individual technique. For instance, the super-resolution techniques (SOFI, SRRF, and deconvolution) record small pixels but typically illuminate longer to reach a certain SNR. On the contrary, Imaging FCS uses larger pixels to be able to record fast for the same reason. Therefore, in combinations of super-resolution and Imaging FCS one typically acquires at the best spatiotemporal resolution and then bins in time or space for super-resolution microscopy or FCS, respectively, to reach a sufficient SNR.

Using the strategy described earlier, both SOFI and SRRF have been combined with Imaging FCS on a sample of LifeAct-labeled actin fibers. This allowed correlating the dynamics measured by Imaging FCS to the better localized actin fibers, providing information how LifeAct interacts with actin^18^. Interestingly the FCS data could also be used to remove artefacts from SRRF images via the dynamics data. FCS has also been combined with deconvolution microscopy^47^ which is a computational technique aimed at reversing the blurring effects of the point spread function of the microscope on the images.

The advantage of computational super-resolution techniques is that they can use the same data as FCS and thus do not need any specialized equipment. This makes these combinations immediately accessible without any modifications. On the other hand, experimental super-resolution techniques, despite requiring specialized equipment, typically reach better spatial resolution. But not all modalities can be readily combined with FCS. Although FCS has single-molecule sensitivity, it measures at concentrations much higher than what would be acceptable for single-molecule localization microscopy techniques. While this can be overcome by using photoswitchable dyes and recording in two colors, one at high, one at very low concentration, as has been demonstrated in the combinations of FCS with single particle tracking (SPT)^67^, it is difficult to do that in a single color.

### Advances in data-handling and data processing in FCS

Developments in optical instrumentation in the last three decades described in the sections above leading to improvements in FCS went together with improvements in the field of computing. Computing power has roughly doubled every two years since the seventies^68^ as predicted by Moore’s law. The use of a data-parallel approach in FPGAs^69^ and later GPUs in multiplexed FCS has led to a significant reduction in the time taken to calculate and fit the autocorrelation functions^35,47,70^ since the same function can be evaluated in every processing element of the GPU. In a further boost to faster evaluation, currently direct camera readout strategies enable numerical analysis while the data is being recorded^71^. As a result, we are at a stage where an experimenter potentially generates data at the rate of hundreds of MB/s. Number-crunching operations on this mammoth dataset is complete within minutes and the experimenter is faced with the complex task of choosing a suitable theoretical model to fit the autocorrelation data. Some of the approaches to fit the data in FCS include non-linear least squares or maximum entropy based fitting routine (MEMFCS)^72^. The choice of a suitable fitting model is typically parameterized as a classification problem in computation.

One of the ways of solving this classification problem in FCS is using Bayesian approaches^73–77^. The utility of other machine learning classifiers including multilayer perceptron, random forests, and support vector machines in FCS are under investigation^78^. In the next section, we describe how deep learning widely used in fluorescence microscopic image processing^79–81^ is utilized for various applications in FCS.

### Use of convolutional neural networks in FCS

**Fig. 3:**
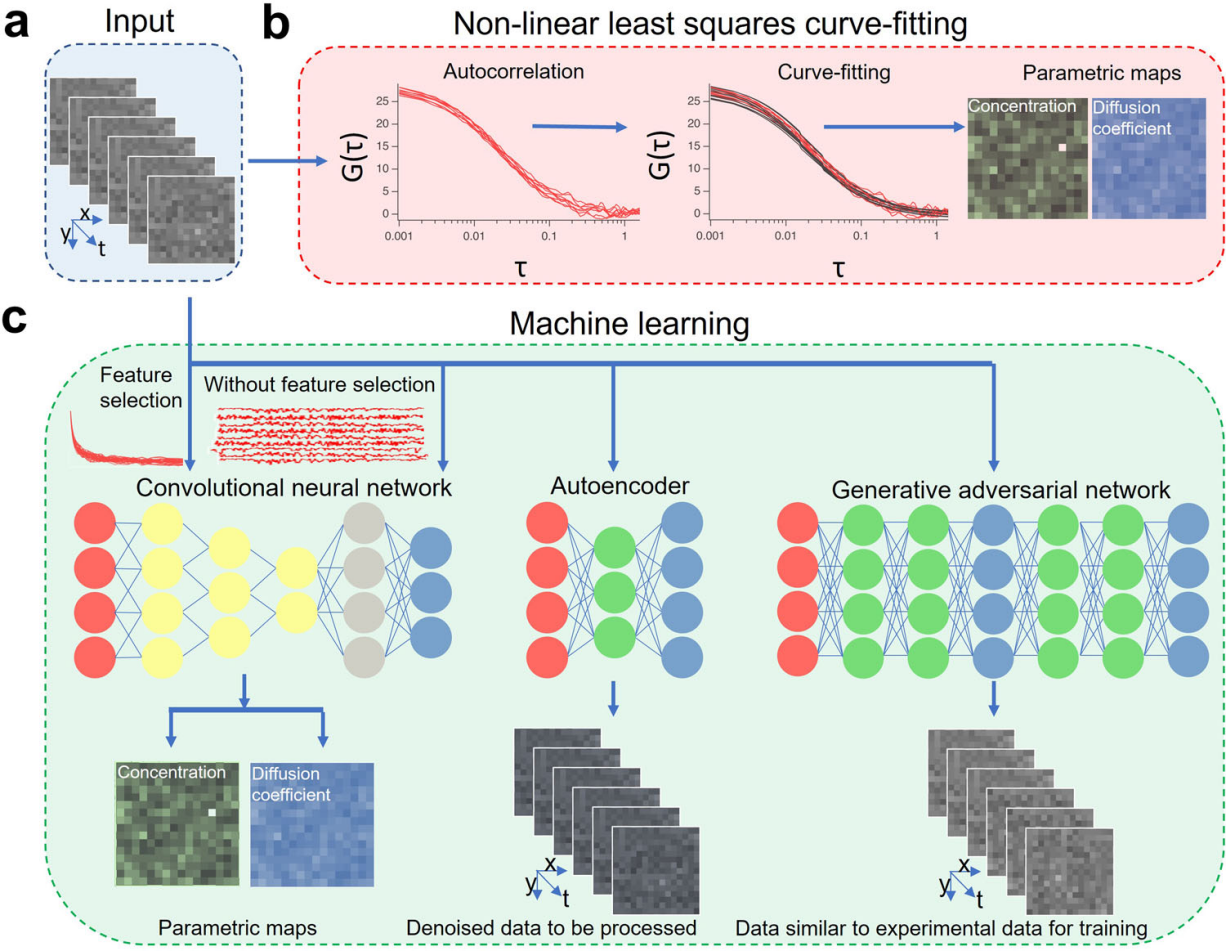
Machine learning in FCS. **a** The input in Imaging FCS is an image stack. **b** In conventional non-linear least squares fitting approach, the autocorrelations are calculated and then fitted to a theoretical model. The spatially resolved physicochemical parameters are the output from this approach. **c** In the machine learning approach, there are two possibilities to estimate the physicochemical parameters. Autocorrelations are calculated and used as a feature selection method. They are used to train a convolutional neural network. Otherwise, the raw intensity is used to train a convolutional neural network. After successful training, when any image stack is given as an input to the network, the parametric maps are obtained as output. Another network architecture relevant for Imaging FCS data analysis is the autoencoder. Autoencoders are used for data-compression and denoising. One of the greatest challenges of using machine learning for FCS is the lack of training data. One solution to generate training data is to use a generative adversarial network. These are two neural networks working together to generate data similar to the one given as an input. In all three types of architectures, the input neurons are shown in red, the output neurons are shown in blue, the convolution neurons are shown in yellow, and the hidden neurons are shown in gray.

Although publications are sparse, the most common deep learning network architecture used in FCS are Convolutional Neural Networks (CNNs)^78,82–89^. CNNs are powerful analysis tools applied to image and time-series analysis (Fig. 3). The major uses of CNNs in FCS are to decide the fitting model and to estimate the kinetic and mobility parameters. Hence CNNs for FCS are designed to solve classification or regression problems in a supervised manner. Unsupervised learning approaches to cluster data to understand its inherent structure in FCS remain to be explored.

Briefly, CNNs consist of multiple layers, each of which performs a convolutional operation with a defined kernel and variable weights. Data sets are propagated through the CNN and the output is compared to a desired outcome or ground truth. The weights are then adjusted in defined ways to reduce the difference between the CNN’s output and the ground truth. This operation is repeated with a large training set so that the CNN gradually learns the properties of the data set. There are a number of different CNN architectures but possible starting points to construct CNNs for FCS are ResNet^90^ and Inception networks^91^ which can be used to construct deep networks and can evaluate multiple scales in parallel, respectively, to reach a good approximation for FCS. ResNet, for instance, has been used in the construction of U-Net, which is widely used in super-resolution and image segmentation.

The universal approximation theorem states that with sufficient learning, neural networks can approximate mathematical functions to a certain level of accuracy and precision^92^. Hence deep CNNs are suitable to learn the autocorrelation function from image stacks. The number of layers in the CNNs determine the depth of the CNN. An increase in the number of layers leads to an increased demand for computational resources (memory and time). Hence one must maintain a delicate balance between efficiency and computational demands while designing CNNs suitable for FCS.

While training the CNN, one must exercise caution not to overfit the data. Overfitted networks perform well only for the training data and not for the test data. Overfitting typically occurs when the size of the training dataset is small. Training on very small datasets leads to over-parameterization of the input data and the network tends to memorize the input and the behavior. Hence the training data must be large enough encompassing a large variety of experimental scenarios to sufficiently train the network. As such the first step in any supervised machine learning paradigm is to obtain labeled ground truth data for training.

However, obtaining sufficient data with the corresponding ground truth data in FCS for training is difficult for at least two reasons. First, it is very time consuming to acquire a large set of FCS data that covers the full range of applicable parameters, including diffusion coefficients, concentrations, and signal-to-noise ratios among others. Second, while accurate estimation of diffusion coefficients of standard samples was made possible by 2f-FCS^93^, standard samples do not cover the entire range of diffusion coefficients accessible to FCS. Even if measured, the diffusion coefficients will be estimated from non-linear least squares fit of a model to the autocorrelation function which has an inherent error associated with the procedure. Although not impossible^94^, creating experimental training sets would suffer from incomplete parameter coverage and inherent errors of the measurements system in addition to time-consuming experiments that need to be repeated for different instruments and conditions, and – due to the required fitting of the data – is model-dependent.

Instead, one can resort to simulations, with parameters chosen that are matched as close as possible to the experimental parameters^95^. In this way, the creation of the training set becomes an automated task. Since any experimental or theoretical illumination and detection geometry and any dynamic processes can be simulated, the CNN becomes (fit) model independent. This way evaluations can be achieved even in cases where no closed-form solution of the correlation function exists, and non-linear least-squares fitting is either not possible or can only be achieved with numerical models that are very time-consuming to evaluate. One should note, though, that a suitable noise model must be incorporated into the simulations to represent experimental conditions. The use of simulations for training has been verified in FCS^83–86^ and SPT^79^. Some software currently available to perform simulations (reviewed here^96^) are SimFCS^24^, PAM^97^, and Imaging FCS^98,99^. Since CNNs are data-hungry, one of the strategies is to simulate data on the fly without saving it to reduce memory requirements.

The simulations for FCS^100,101^ are modeled on the fundamental physics of diffusion which is a stationary process. The presence of deviations from stationarity (for instance due to photobleaching or inhomogeneities) in the experimental data will lead to errors of the estimates of physicochemical parameters from the CNNs. Two different strategies can be followed here. Either the simulated training sets include these non-stationary processes, or the experimental data must be corrected (for instance by bleach correction) before being evaluated by CNN.

One of the factors determining the precision of estimates recovered from NLS fitting of FCS data is the length of the time-series of the image stack. Empirically, it was shown that to obtain at most a 20% coefficient of variation^101^ (standard deviation/mean), the measurement time needs to be at least a hundred times the diffusion time of the molecule being investigated. However, we can train CNNs with many such shorter image-stacks which are typically not sufficient for a good NLS fit. By training the CNN on a large set of these small data sets over a wide parameter range we can obtain a CNN that fits data at significantly reduced measurement time, which an NLS fit could not properly evaluate.

CNNs have been trained on FCS data in two different ways so far^83–87^ (Fig. 3). Either the CNN is trained on raw imaging data and thus learns the dynamics directly from the intensity traces or the CNN is trained on pre-computed features, i.e., in our case the correlation functions of interest. The advantage of using computed features is that this approach preselects features for training reducing the dimensionality of the search space to perform the optimization. For instance, while training for FCS, one could train the neural networks on the raw images or on the pixelwise autocorrelation functions. Assuming 50,000 frames of 128 × 128 images stored in 16-bit format are used, the size of one training data is ~2 GB. Instead, if one uses the autocorrelation function, which has generally <1000 points, as a pre-computed feature, the size of the training dataset is ~65 MB, corresponding to a reduction of training data size by a factor 25.

Both strategies have their advantages. While using the correlation functions as training sets, the much smaller size of the test data leads to smaller, more compact CNNs which do not impose huge demands on the computational infrastructure. The use of the raw images, on the other hand, has the advantage that it is not limited by the temporal averaging performed by the correlation functions. One of the other advantages of using the raw imaging data is to avoid sampling artefacts due to the multi-tau algorithm used to calculate the autocorrelation function^7,100,102^

CNNs have the potential to address several limitations of FCS. First, the measurement time required is dictated by the fact that the autocorrelation function in FCS is a biased estimator^103^ and it converges only at long measurement times^101,104^. Once properly trained, CNNs learn the bias and produce more accurate and precise parameter estimates even when using less data^83–87^.

Second, FCS resolution is limited in distinguishing multiple particles with different diffusion times. Using conventional non-linear least squares fitting on FCS data where the SNR is not a limiting factor and when there is an equal distribution of fast and slow particles, the ratio of the faster particle’s diffusion coefficient to that of the slower particle’s must be at least 1.6 in order to distinguish them as two different particles^105^. As the proportion or the SNR varies, the minimum ratio of diffusion coefficients necessary to be distinguished as two different diffusing particles increases and can easily exceed a factor 10 in diffusion coefficients (a factor 1000 in mass). CNN-based data analysis^88^ enabled reliable estimation of diffusion coefficients of fast and slow particles even in situations when the ratio of the faster particle’s diffusion coefficient to that of slower particle was <1.6 in simulations.

Third, CNN-based analysis also provides considerable improvement in the time taken to estimate the parameters. Sufficiently trained CNNs are faster than the iterative non-linear least square fits which require multiple rounds of calculation to reach the optimal value. This becomes even more evident in cases where no analytical solution is available and numerical models need to be employed^95^. Fourth, the knowledge gained by any trained network on free diffusion also has the potential to be adapted for novel but similar tasks (referred to as transfer learning). Transfer learning for a similar task is faster to train when compared to training a network ab-initio for the same task. For instance, by transfer learning, networks trained for obtaining mobility estimates could be utilized for spatial investigations such as presence of diffusion inhomogeneities. Fifth, the effects of systemic behavior affecting the autocorrelation functions (large aggregates passing through the detection area, sample movement, or photobleaching) can be mitigated using deep learning. In principle, networks can be trained to correct for these artefacts and thus lead to more robust and simpler FCS systems.

CNNs have the potential to be used in the classification of the type of diffusion. In biological samples diffusion is often not free as biomolecules move in a complex matrix and have many opportunities to interact with their surroundings specifically or non-specifically. This is often determined by measuring the diffusion coefficient of a sample in dependence on the area observed, a procedure commonly referred to as diffusion law analysis^106^. Briefly, if one changes the size of the observation area, e.g., by binning adjacent pixels in Imaging FCS, or by changing the laser spot size in a confocal or STED microscope^54,107^, the average time taken by molecules undergoing free diffusion to traverse the areas of different size increase linearly with observation area. Any deviations from linearity imply different diffusion modes, which can subsequently be identified from the type and strength of the deviation^106^. Without calculating and fitting the autocorrelation at different length scales as performed in FCS diffusion law analysis, the spatiotemporal information could be directly extracted from a raw image stack. As a result, CNNs can be trained on raw image stacks of simulations of various diffusion modes and hence can predict the diffusive modes of the particle.

Apart from use of CNNs to classify diffusion, CNNs also have the potential to be used in number and brightness (N&B) analysis^108,109^. N&B analysis is an offshoot of FCS providing only the aggregation and concentration profiles of the dynamic system under investigation. Typically, in N&B analysis, the observed intensity of the pixel is deconstructed mathematically to reveal the contributions of the concentration of the fluorophore and of the aggregation state of the fluorophore. Current mathematical methods are not efficient in deconstructing the aggregation states when multiple aggregation states of the same molecule are present at the same time. CNNs modeled as a regression problem have the potential to improve the deconvolution of the aggregation states in N&B analysis.

Apart from CNNs, other deep learning architectures which have potential for FCS are autoencoders^110^, generative adversarial networks^111^, and mixture density networks^112^. Mixture density networks hold great promise in estimating the parameters of combinations of probability distributions similar to the MEMFCS^72^ approach.

As the name suggests autoencoders learn the important characteristics of the input data and yield a lean representation of the same data. Typically used for dimensionality reduction, autoencoders serve as a denoiser for the raw data. Hence FCS data have the potential to be denoised using an autoencoder before being used for data analysis.

Generative adversarial networks are another class of neural networks which consist of two networks working in tandem. These are typically used to create datasets similar to the dataset given as an input. The first ‘generative’ network creates the dataset while the next ‘adversarial’ network discriminates whether the created dataset is similar or dissimilar to the original dataset. This is very useful for generating data for training with similar noise structures to the experimental data used as an input. This approach overcomes the need to have an analytical noise model to create simulation data for training.

### Power and current limitations in live tissues

A field that stands to gain considerably by FCS advances involving super-resolution microscopy and machine learning are measurements in live tissues and organisms. Measurements of cell autonomous processes can be conducted in cell cultures, at least qualitatively. But even here, there are indications that quantitative results differ between cell and in vivo measurements. Consequently, there have been a number of investigations in various model organisms, albeit at a much lower number than FCS applications in cells or in vitro. Today, FCS has mainly been used to study transport^45,46,75,98,113–130^, binding^131–134^, and organization^135^ in live organisms including fish (reviewed here^136–138^), worms, and flies (Table 1).

**Table 1:** FCS applications in vivo.

Model organism	Technique	Refs.
Zebrafish	FCS	^75,113–120,142^
	Scanning FCS	^126–128^
	FCCS	^131–134,143^
	Imaging FCS	^45,98,125,135^
Nematode	FCS	^122,123^
	Two photon FCS	^129^
	Scanning FCS	^129^
	Imaging FCS	^46^
Fruit fly	FCS	^114,121,122,144^

The major advantage of FCS in live tissues is the power to quantify the dynamics of various biological processes in the presence of all relevant interaction partners, which can significantly influence biomolecular interactions, and under physiological environmental conditions. However, the use of FCS in live tissues has been hampered by various challenges. First, tissue movement cannot be avoided under all circumstances (for examples, tissue development and growth, heartbeat, or blood flow) distorting correlation functions and making data evaluation unreliable. Second, measurements in live tissues often have higher background and thus do not have a SNR as high as those in cells or purified systems and thus limiting measurement accuracy. And spherical aberration due to index heterogeneity in the tissue further reduce resolution and SNR.

New developments are addressing at least some if not all these issues. Direct camera-readout^71^ and online analysis of FCS data enables identification of movement artefacts enabling user intervention and avoiding unnecessary data loss. Alternatively, while analyzing data from live tissues containing anomalies including drifts, spikes, or unwanted fluctuations^103^, one can apply a recently developed theoretical framework utilizing temporal segmentation. It remains to be tested if machine learning can aid in the analysis of data with distortions. Second, it has been shown that CNNs can be constructed that require less data to obtain the same parameter accuracy and better precision compared to standard non-linear least-squares curve fitting. Finally, adaptive optics shows great promise to improve data acquisition in complex environments with heterogenous refractive index distributions^139,140^. Coupled with super-resolution and FCS this could lead to new accuracy and precision of spatiotemporal events even in complex environments.

### Outlook

FCS has been widely used to study diffusion and binding in biological systems. However, in its classical confocal modality, FCS has been limited in multiple ways. It provided high intrinsic resolution of molecular dynamics but was diffraction-limited in its analysis. It provided only a single spot or at best a few spots for measurements and did not provide a contiguous image of a sample, making integration with imaging modalities difficult. It required long measurement times to calculate correlations that could be evaluated with sufficient accuracy and precision. Data analysis was also complicated due to a variety of factors including the inhomogeneity of the errors of the correlation functions, the fact that correlation function models could be calculated only for the simplest cases when applying appropriate approximations, the choice of the model for the fitting function and finally the large number of calculations required for multiplexed approaches.

Many of these challenges have been met or are being addressed. The use of STED-FCS achieved sub-diffraction resolution for FCS. Later the use of fast and sensitive array detectors in FCS enabled multiplexing. Online evaluation of sub-micrometer spatially resolved structural data and sub-millisecond temporally resolved dynamic data in a variety of biological samples is already possible and parallel processing of data using GPUs with multiple computing elements has dramatically reduced the time taken for data-evaluation. The use of machine learning in FCS promises user autonomous, model-independent data analysis with increased time-resolution. The combination of FCS and super-resolution, deep learning, and the use of more powerful sensors^141^ permits creating new imaging modalities that provide spatial and temporal information on molecular processes with unprecedented resolution in real-time and at a repetition rate of seconds. In combination with computational super-resolution techniques these modalities do not require specialized equipment. The integration of FCS and super-resolution microscopy promises to provide unprecedented spatiotemporal resolution in biological samples, even in vivo. The implementation of machine learning algorithms in combination with GPU processing will allow real-time data analysis, render data evaluation model-independent, and simplify experiments, making these powerful techniques available to non-expert users.

### Box 1 – Important developments and future challenges of FCS

#### Important developments

- FCS in its various forms and its hyphenated techniques with super-resolution microscopy allows long-term observations of various biological processes across a variety of biological scales including cells, tissues, organoids, and whole organisms providing complementary information on dynamics and structure within a single measurement.
- The introduction of online evaluation and GPU-based parallel computing in FCS has led to a reduction in the time taken to analyze the data. The use of machine learning in FCS promises model-independent data analysis. This is especially important in cases where currently no analytic fit function is available.

#### Future challenges

- Today, deep learning has the capacity to simplify and accelerate data evaluation for the user making FCS more applicable and easier to use for the wider biological community. Which network architectures in deep learning are useful for FCS analysis is an open question and will need to be addressed in the future. Extensive benchmarking and stress testing needs to be performed to understand the advantages of deep learning over conventional regression approaches including NLS fitting. The use of explainable AI or interpretable AI in FCS will lead to a better comprehension of the decision making of AI while estimating the parameters in FCS.
- The combination of FCS with super-resolution has combined dynamics with structure. The next frontier to tackle is to combine functional assays with FCS and super-resolution. From a single measurement, one will then be able to obtain a spatially resolved map of structure, function, and dynamics.
- Bringing these advances into live tissues and organisms will be a major challenge. It will allow obtaining dynamics, structure, and interaction maps in physiologically relevant environments within a single measurement in real time. The simultaneous measurement of these parameters will provide new information and insights not available to date.

### Reporting summary

Further information on research design is available in the Nature Portfolio Reporting Summary linked to this article.

## Supplementary information

Reporting Summary
